# Beta-Estradiol Regulates Voltage-Gated Calcium Channels and Estrogen Receptors in Telocytes from Human Myometrium

**DOI:** 10.3390/ijms19051413

**Published:** 2018-05-09

**Authors:** Adela Banciu, Daniel Dumitru Banciu, Cosmin Catalin Mustaciosu, Mihai Radu, Dragos Cretoiu, Junjie Xiao, Sanda Maria Cretoiu, Nicolae Suciu, Beatrice Mihaela Radu

**Affiliations:** 1 Department of Anatomy, Animal Physiology and Biophysics, Faculty of Biology, University of Bucharest, Splaiul Independentei 91-95, 050095 Bucharest, Romania; adela.banciu79@gmail.com (A.B.); danieldumitrubanciu@gmail.com (D.D.B.); 2 Faculty of Medical Engineering, University Politehnica of Bucharest, Gheorge Polizu Street 1-7, 011061 Bucharest, Romania; 3 Department of Life and Environmental Physics, Horia Hulubei National Institute of Physics and Nuclear Engineering, Reactorului 30, P.O. Box MG-6, 077125 Magurele, Romania; cosmin@nipne.ro (C.C.M.); mradu@nipne.ro (M.R.); 4 Faculty of Applied Chemistry and Materials Science, University Politehnica of Bucharest, 011061 Bucharest, Romania; 5 Department of Cell and Molecular Biology and Histology, Carol Davila University of Medicine and Pharmacy, 050474 Bucharest, Romania; dragos@cretoiu.ro (D.C.); sanda@cretoiu.ro (S.M.C.); 6 Alessandrescu-Rusescu National Institute of Mother and Child Health, Fetal Medicine Excellence Research Center, 020395 Bucharest, Romania; nsuciu54@yahoo.com; 7 Cardiac Regeneration and Ageing Lab, Experimental Center of Life Sciences, School of Life Science, Shanghai University, Shanghai 200444, China; junjiexiao@shu.edu.cn; 8 Department of Obstetrics and Gynecology, Polizu Clinical Hospital, 011062 Bucharest, Romania; 9 Life, Environmental and Earth Sciences Division, Research Institute of the University of Bucharest (ICUB), 91-95 Splaiul Independenţei, 050095 Bucharest, Romania

**Keywords:** beta-estradiol, human uterine myometrium, telocytes, calcium signaling, voltage-gated calcium channels, estrogen receptors

## Abstract

Voltage-gated calcium channels and estrogen receptors are essential players in uterine physiology, and their association with different calcium signaling pathways contributes to healthy and pathological conditions of the uterine myometrium. Among the properties of the various cell subtypes present in human uterine myometrium, there is increasing evidence that calcium oscillations in telocytes (TCs) contribute to contractile activity and pregnancy. Our study aimed to evaluate the effects of beta-estradiol on voltage-gated calcium channels and estrogen receptors in TCs from human uterine myometrium and to understand their role in pregnancy. For this purpose, we employed patch-clamp recordings, ratiometric Fura-2-based calcium imaging analysis, and qRT-PCR techniques for the analysis of cultured human myometrial TCs derived from pregnant and non-pregnant uterine samples. In human myometrial TCs from both non-pregnant and pregnant uterus, we evidenced by qRT-PCR the presence of genes encoding for voltage-gated calcium channels (Cav3.1, Ca3.2, Cav3.3, Cav2.1), estrogen receptors (ESR1, ESR2, GPR30), and nuclear receptor coactivator 3 (NCOA3). Pregnancy significantly upregulated Cav3.1 and downregulated Cav3.2, Cav3.3, ESR1, ESR2, and NCOA3, compared to the non-pregnant condition. Beta-estradiol treatment (24 h, 10, 100, 1000 nM) downregulated Cav3.2, Cav3.3, Cav1.2, ESR1, ESR2, GRP30, and NCOA3 in TCs from human pregnant uterine myometrium. We also confirmed the functional expression of voltage-gated calcium channels by patch-clamp recordings and calcium imaging analysis of TCs from pregnant human myometrium by perfusing with BAY K8644, which induced calcium influx through these channels. Additionally, we demonstrated that beta-estradiol (1000 nM) antagonized the effect of BAY K8644 (2.5 or 5 µM) in the same preparations. In conclusion, we evidenced the presence of voltage-gated calcium channels and estrogen receptors in TCs from non-pregnant and pregnant human uterine myometrium and their gene expression regulation by beta-estradiol in pregnant conditions. Further exploration of the calcium signaling in TCs and its modulation by estrogen hormones will contribute to the understanding of labor and pregnancy mechanisms and to the development of effective strategies to reduce the risk of premature birth.

## 1. Introduction

Uterine contractions represent a key point throughout pregnancy. Considered to be normal during pregnancy, uterine contractions may also be responsible for triggering premature or dysfunctional labor. During pregnancy, the myometrium suffers morphological and adaptive changes which enable it to become a forceful organ necessary for delivery. Myometrium contractility involves, among other physiological mechanisms that produce excitation in the uterus, changes in Ca^2+^ signals. Smooth myofiber contraction requires actin and myosin myofilaments and their interaction. The cross-bridge formation and contraction are mediated by elevated levels of intracellular Ca^2+^ and myosin light-chain phosphorylation.

Calcium signaling plays an essential role in uterine contractility and involves several key players, including voltage-gated calcium channels, calcium-activated chloride channels, large conductance Ca^2+^-activated K^+^ channels (BK(Ca)), calcium-sensing receptors, and transient receptor potential channels [1,2,3,4,5,6]. On the basis of their activation electrophysiological features, voltage-gated calcium channels have been classified in high-voltage-activated Ca^2+^ channels (HVA) and low-voltage-activated Ca^2+^ channels (LVA). Among the HVA channels, only the L-type Ca^2+^ channels are expressed in uterus. Their family includes Cav1.1 (*CACNA1S* gene), Cav1.2 (*CACNA1C* gene), Cav1.3 (*CACNA1D* gene), and Cav1.4 (*CACNA1F* gene) channels. Meanwhile, in uterus, the LVA channels are represented by T-type calcium channels, classified as Cav3.1 (*CACNA1G* gene), Cav3.2 (*CACNA1H* gene), and Cav3.3 channel (*CACNA1I* gene).

In uterine contractility, intracellular Ca^2+^ comes from two sources: entry across the sarcolemma through voltage-gated L-type Ca^2+^ channels and release from the sarcoplasmic reticulum [7]. In the uterus, the major Ca^2+^ source for contraction comes from the extracellular space through voltage-gated L-type Ca^2+^ channels [8]. Indeed, calcium influx through the L-type Ca^2+^ channels was demonstrated to be essential for labor and uterine contractility [1]. Moreover, uterine phasic contractions were abolished if the L-type Ca^2+^ channels were blocked [1]. In addition, the T-type Ca^2+^ channels might also contribute to calcium entry in smooth myocytes in human myometrium [9].

The estrogen 17beta-estradiol was demonstrated in different types of tissue (e.g., neuronal, uterine etc.) to modulate multiple components of the calcium transport pathways, including BK(Ca) [10], small conductance Ca^2+^-activated potassium channel subtype 3 (SK3, [11]), HVA channels [12,13], LVA channels [14], Na^+^/Ca^2+^ exchanger (NCX, [15]), transient receptor potential channels [16,17], etc. In particular, beta-estradiol and its receptors play an essential role in pregnancy and labor [18,19,20] and were shown to act on uterine myometrium by means of various calcium signaling pathways.

Telocytes (TCs) have been described by our team as a new cellular type in human myometrium [21]. Characterized by extremely long telopodes, TCs were described in the interstitial space of numerous organs, as 3-D network-forming cells by homocellular or heterocellular contacts [22,23]. It was shown that TCs release extracellular vesicles, such as exosomes and ectosomes, to regulate the functions of the surrounding cells in non-pregnant and pregnant myometrium [24]. The gene profiles, proteome and secretome features of TCs were recently revealed [25,26,27]. Moreover, our early studies showed that TCs express estrogen and progesterone receptors, which indicates them as potentially responsible for myogenic contractility modulation under hormonal control [28].

There are some extensive reviews describing the main morphological features and possible functions of TCs in reproductive organs [29,30,31,32]. One of the most important aspects relates to the TCs stemness capacity which might contribute to regeneration and repair processes, as it has been previously shown [33]. In particular, a recent review highlights the contribution of calcium signaling in interstitial cells (including TCs, interstitial cells of Cajal, interstitial Cajal-like cells) to uterine, cardiac, and urinary physiology and pathology [34].

Recently, we demonstrated, by immunofluorescence and electrophysiology, the presence of the T-type calcium channels in human uterine myometrial TCs [35]. Our studies also confirmed the antagonistic pharmacological effect of mibefradil on voltage-gated calcium currents in TCs from human uterine myometrium [35] and its modulatory effect on telopodes growth after stimulation with near-infrared low-level lasers [36]. We also evidenced, by patch-clamp recordings, the presence of HVA calcium currents in TCs from human non-pregnant myometrium [35]. To date, no studies have yet quantified the mRNA levels encoding the voltage-gated calcium channels in human uterine myometrial TCs. Moreover, despite extensive studies describing the effect of beta-estradiol on human uterine myometrium, no description of its mechanism of action on TCs was done.

TCs were recently detected in human uterine leiomyoma in higher number compared with the areas of adjacent fibrotic and normal myometrium, suggesting that it may provide a scaffold for newly formed myocytes or control important downstream signaling pathways [37]. By contrast, another study claims that TCs are absent inside leiomyomas and represent ~2% of the cells in the normal myometrium [38]. Since leiomyomas are considered estrogen-dependent tumors, and leiomyoma tissue is more sensitive to estradiol (has more estrogen receptors in comparison to normal myometrium), authors suggested that the loss of the TC network might lead to myocytes taking up the hormonal sensor function, which can determine an uncontrollable proliferation of myocytes [38]. However, in our opinion, both studies have a major weakness since they do not demonstrate the presence of TCs with the aid of their most specific immunohistochemical markers CD34 and platelet-derived growth factor receptor (PDGFR) α or β [39,40], but use c-kit for the detection. TCs’ c-kit positivity is also used by the authors to suggest the role of TC progenitor cells for the development of leiomyoma. Moreover, on the basis of the assumption that TCs are involved in angiogenesis [41,42], they also suggest that TCs loss may be responsible for decreased vessel formation within the myometrium and subsequent shifting from aerobic to anaerobic metabolism in smooth muscle cells [38]. The subsequent hypoxia and the decreased angiogenesis represent crucial factors for leiomyoma development [43,44].

Uterine TCs have been reported to be present in different reproductive states; while endometrial TCs have been hypothesized to be involved in glandular support and stromal cell communication, myometrial TCs were considered to be responsible for the initiation and propagation of contractile activity [45]. Calcium is a key player in uterine physiological activity, and multiple calcium signaling pathways have been evidenced to be activated in pregnancy and labor. In particular, voltage-gated calcium channels (e.g., HVA and LVA calcium channels) [46,47] or estrogen receptors [48] have been described to be involved in the spontaneous contractile activity in both pregnant and non-pregnant uterus. Therefore, our results in human myometrial TCs from pregnant and non-pregnant uterus are clinically relevant.

Our goal was to determine the level of expression of voltage-gated calcium channels and estrogen receptors in human myometrial TCs and to evidence potential differences in pregnant versus non-pregnant uterus. Additionally, we focused our attention on the modulatory effect of female sex hormones (e.g., beta-estradiol) exerted on the voltage-gated calcium channels and estrogen receptors in human myometrial TCs from pregnant uterine samples.

Our study is trying to highlight some of the calcium signaling mechanisms associated with voltage-gated calcium channels and estrogen receptors activation and to understand their involvement in the pregnancy state.

## 2. Results

### 2.1. Pregnancy Induces Changes in mRNA Levels Encoding the Voltage-Gated Calcium Channels Compared to the Non-Pregnant Condition in Human Uterine Myometrial TCs

**Figure 1:**
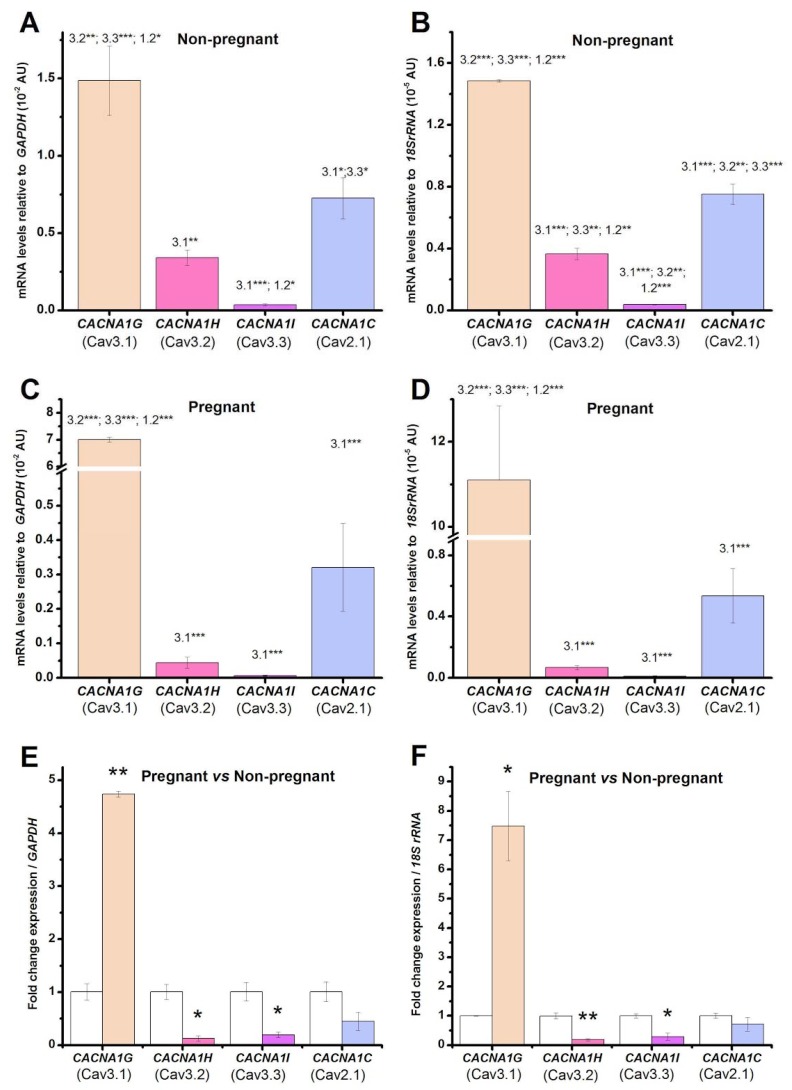
mRNA levels encoding the voltage-gated calcium channels in human myometrial telocytes (TCs) from non-pregnant (**A**,**B**) and pregnant (**C**,**D**) uterine samples. The mRNA levels for *CACNA1G* (Cav3.1 channel), *CACNA1H* (Cav3.2 channel), *CACNA1I* (Cav3.3 channel), and *CACNA1C* (Cav1.2 channel) were normalized against two different reference genes, *GAPDH* (**A**,**C**) and *18S rRNA* (**B**,**D**), and were plotted as mean ± SD (*N* = 3), corresponding to cell batches extracted from either pregnant or non-pregnant uterine myometrium. One-way ANOVA analysis, applied for the samples derived either from pregnant uterus or from non-pregnant uterus, indicated: (**A**) F = 78, *p* < 0.001; (**B**) F = 545, *p* < 0.001; (**C**) F = 2580, *p* < 0.001; (**D**) F = 77, *p* < 0.001. The post-hoc Bonferroni test was applied, and the statistically significant pairs are indicated by the numbers corresponding to the channels symbols, as follows: Cav3.1, 3.2, 3.3, and 1.2; (**E**,**F**) Fold-change for each voltage-gated calcium channel between non-pregnant and pregnant uterine samples, where non-pregnant samples were defined as calibrator, considering as housekeeping gene *GAPDH* (**E**) and *18S rRNA* (**F**), respectively. Statistical significance is indicated with asterisks (* 0.01 < *p* < 0.05; ** 0.001 < *p* < 0.01; *** *p* < 0.001).

In a previous study, we demonstrated the immunopositivity for T-type calcium channels (i.e., Cav3.1 and Cav3.2) in human myometrial TCs from pregnant and non-pregnant uterus [35]. We also evidenced the presence of T-type calcium currents and HVA currents in human myometrial TCs by employing patch-clamp recordings [35]. In the present study, we wanted to quantify the mRNA levels for voltage-gated calcium channels by qRT-PCR in both types of uterine samples. Indeed, we confirmed the presence of the followings genes: *CACNA1G* (encoding Cav3.1 T-type calcium channel), *CACNA1H* (encoding Cav3.2 T-type calcium channel) > *CACNA1I* (encoding Cav3.3 T-type calcium channel), and *CACNA1C* (encoding Cav1.2 L-type calcium channel) in TCs from both pregnant and non-pregnant uterine myometrium. The ranking of the mRNA levels encoding the voltage-gated calcium channels relative to *GAPDH* was: *CACNA1G* (Cav3.1) > *CACNA1C* (Cav1.2) > *CACNA1H* (Cav3.2) > *CACNA1I* (Cav3.3) in TCs from both pregnant (Figure 1C,D) and non-pregnant (Figure 1A,B) uterine myometrium. The same mRNA level ranking was obtained relative to the other housekeeping gene *18S rRNA*.

We also evaluated if there were any changes in the mRNA levels encoding the voltage-gated calcium channels due to the pregnancy condition (Figure 1E,F). When considering *GAPDH* as a reference gene and the non-pregnant condition as a calibrator, *CACNA1G* was upregulated 3.74-fold (*p* < 0.01), while *CACNA1H*, *CACNA1I*, and *CACNA1C* were downregulated 0.87-fold (*p* < 0.05), 0.81-fold (*p* < 0.05), and 0.56-fold (not significant), respectively, in pregnant samples versus non-pregnant samples. On the other hand, when considering *18S rRNA* as a reference gene and the non-pregnant condition as a calibrator, *CACNA1G* was upregulated 6.48-fold (*p* < 0.01), while *CACNA1H*, *CACNA1I,* and *CACNA1C* were downregulated 0.82-fold (*p* < 0.05), 0.71-fold (*p* < 0.05), and 0.29-fold (not significant), respectively, in pregnant samples versus non-pregnant samples.

### 2.2. Pregnancy Induces Changes in mRNA Levels Encoding the Estrogen Receptors Compared to the Non-Pregnant Condition

**Figure 2:**
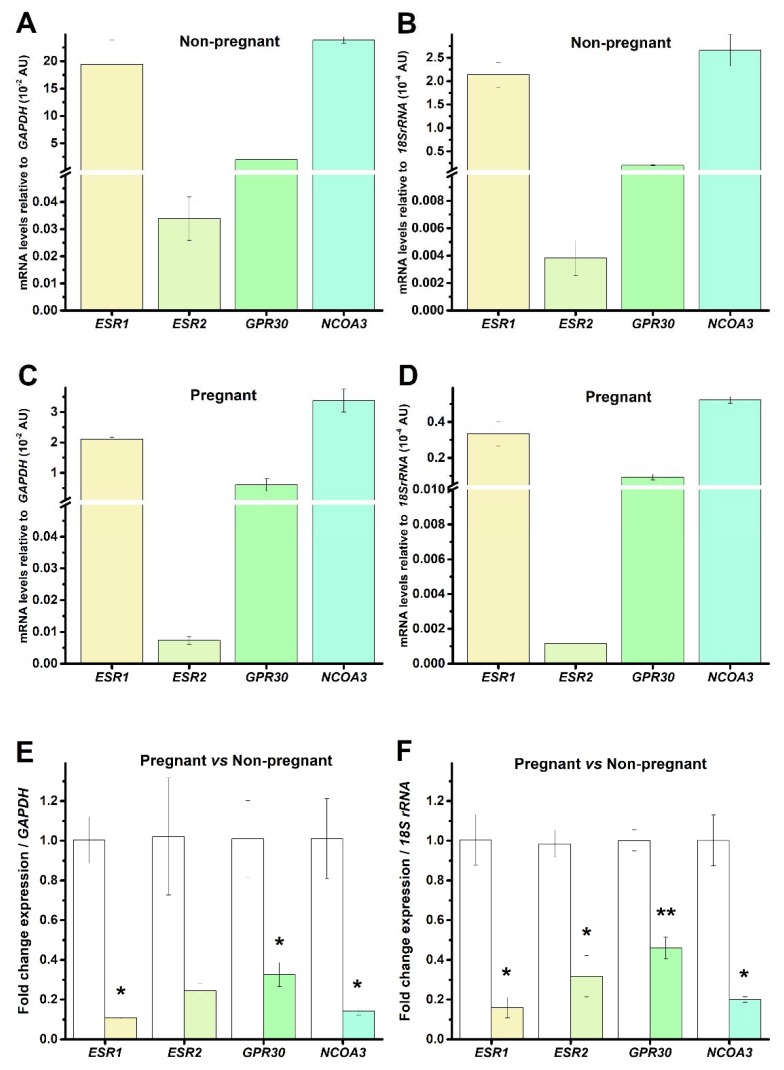
mRNA levels encoding the estrogen receptors and nuclear receptor co-activator in human myometrial TCs from non-pregnant (**A**,**B**) and pregnant (**C**,**D**) uterine samples. The mRNA levels of *ESR1* (estrogen receptor 1), *ESR2* (estrogen receptor 2), *GPR30* (G protein-coupled receptor 30), and *NCOA3* (nuclear receptor coactivator 3), were normalized against two different reference genes, *GAPDH* (**A**,**C**) and *18S rRNA* (**B**,**D**) and were plotted as mean ± SD (*N* = 3), corresponding to cell batches extracted from either pregnant or non-pregnant uterine myometrium. (**E**,**F**) Fold-change for each estrogen receptor in pregnant uterine samples versus the non-pregnant condition, where non-pregnant samples were defined as calibrator, considering as housekeeping gene *GAPDH* (**E**) and *18S rRNA* (**F**), respectively. Statistical significance is indicated with asterisks (* 0.01 < *p* < 0.05; ** 0.001 < *p* < 0.01).

Estrogens (i.e., beta-estradiol) are essential hormones in uterine physiology. They may act on multiple cellular targets, including estrogen receptors (ESR1 and ESR2), G protein-coupled receptors (GPR30), and nuclear receptors (NCOA3). The role of estrogen receptors in the uterine myometrium physiology was demonstrated in different species, including human, canine, equine, rat etc. [49,50,51,52,53]. We might expect that these genes are present in different subtypes of cells of the human uterine myometrium, including TCs and smooth muscle cells. Therefore, we analyzed the presence of *ESR1*, *ESR2*, *GPR30*, and *NCOA3* genes in human myometrial TCs from non-pregnant and pregnant uterine samples. We confirmed the presence of all four analyzed genes, with the following ranking: *NCOA3* > *ESR1* > *GPR30* > *ESR2*. Their presence was not dependent on the pregnancy state or the used housekeeping gene (Figure 2A–D).

We also demonstrated a significant downregulation of the mRNA levels of all four genes in human myometrial TCs from pregnant uterine samples compared to non-pregnant uterine samples (Figure 2E,F). When considering *GAPDH* as a reference gene and the non-pregnant condition as a calibrator, the estrogen receptors were downregulated in pregnant samples 0.89-fold (*p* < 0.05, *ESR1*), 0.77-fold (not significant, *ESR2*), 0.67-fold (*p* < 0.05, *GPR30*), 0.86-fold (*p* < 0.05, *NCOA3*) versus non-pregnant samples. Meanwhile, when considering *18S rRNA* as a reference gene and the non-pregnant condition as a calibrator, the estrogen receptors were downregulated in pregnant samples 0.84-fold (*p* < 0.05, *ESR1*), 0.68-fold (*p* < 0.05, *ESR2*), 0.54-fold (*p* < 0.01, *GPR30*), 0.8-fold (*p* < 0.05, *NCOA3*) versus non-pregnant samples.

### 2.3. Beta-Estradiol Downregulates Voltage-Gated Calcium Channels in TCs from Human Pregnant Uterine Myometrial Cultures

We considered only the pregnant condition for further testing the effect of beta-estradiol. Primary cultures of TCs from human pregnant myometrium were treated for 24 h with increasing concentrations of beta-estradiol (10, 100, and 1000 nM). The treatment protocol with beta-estradiol was established on the basis of previous studies [54]. We quantified by qRT-PCR the mRNA levels encoding the Cav3.1, Cav3.2, Cav3.3, and Cav1.2 channels at different doses of beta-estradiol, considering *GAPDH* or *18S rRNA* as housekeeping genes.

**Figure 3:**
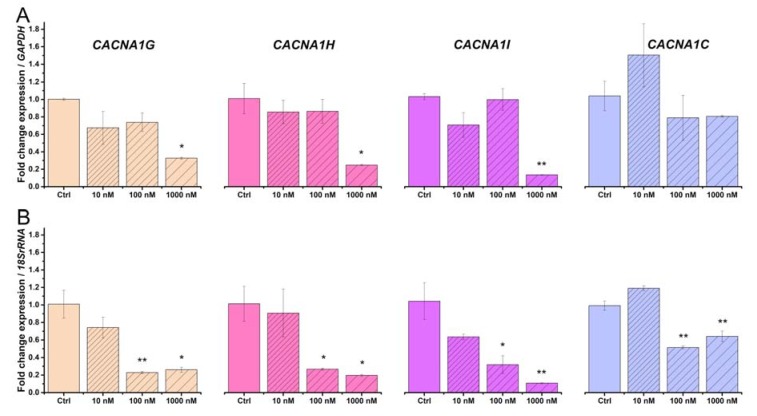
Effect of beta-estradiol treatment (10, 100, and 1000 nM) on the mRNA levels encoding the voltage-gated calcium channels in human myometrial TCs from pregnant uterine samples. Fold-change for each voltage-gated calcium channel upon treatment with beta-estradiol, where the non-treated samples were considered as calibrator. The data were normalized against *GAPDH* (**A**) and 18S rRNA (**B**) and were plotted as mean ± SD (*N* = 3). Statistical significance is indicated with asterisks (* 0.01 < *p* < 0.05; ** 0.001 < *p* < 0.01).

Beta-estradiol treatment downregulated the mRNAs encoding the voltage-gated calcium channels, with distinct patterns for each channel subtype. Considering the untreated cells as calibrator and *GAPDH* as the reference gene, *CACNA1G* (One way ANOVA, F = 13, *p* < 0.05), *CACNA1GH* (One way ANOVA, F = 14, *p* < 0.05), and *CACNA1GI* (One way ANOVA, F = 37, *p* < 0.01) channels were significantly downregulated (Figure 3A). The Bonferroni post-hoc analysis indicated that 100 nM beta-estradiol significantly downregulated *CACNA1G* by 0.67-fold (*p* < 0.05), *CACNA1GH* by 0.75-fold (*p* < 0.05), *CACNA1GH* by 0.86-fold (*p* < 0.05), versus non-treated samples. Considering the untreated cells as calibrator and *18S rRNA* as the reference gene, *CACNA1G* (One way ANOVA, F = 29, *p* < 0.01), *CACNA1GH* (One way ANOVA, F = 12.5, *p* < 0.05), *CACNA1GI* (One way ANOVA, F = 24, *p* < 0.01), and *CACNA1C* (One way ANOVA, F = 103, *p* < 0.001) channels were significantly downregulated (Figure 3B). The Bonferroni post-hoc analysis indicated that 100 and 1000 nM beta-estradiol significantly downregulated *CACNA1G* by 0.77-fold (*p* < 0.01) and 0.74-fold (*p* < 0.05), *CACNA1GH* by 0.73-fold (*p* < 0.05) and 0.82-fold (*p* < 0.05), *CACNA1GH* by 0.68-fold (*p* < 0.05) and 0.89-fold (*p* < 0.01), *CACNA1GC* by 0.49-fold (*p* < 0.01) and 0.36-fold (*p* < 0.01), respectively, versus the non-treated samples. It should be mentioned that the downregulation of *CACNA1GC* mRNA upon treatment with beta-estradiol was statistically significantly only relative to *18S rRNA* and not relative to *GAPDH*.

### 2.4. Beta-Estradiol Downregulates Estrogen Receptors in TCs from Human Pregnant Uterine Myometrial Cultures

**Figure 4:**
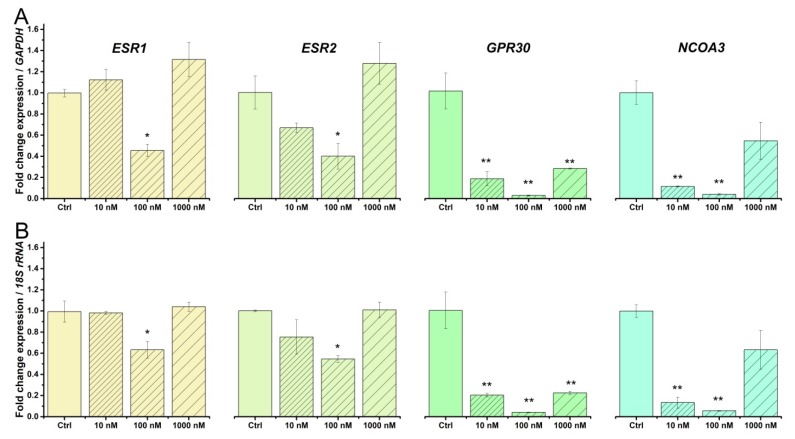
Effect of beta-estradiol treatment (10, 100, and 1000 nM) on the mRNA levels encoding the estrogen receptors and nuclear receptor co-activator in human myometrial TCs from pregnant uterine samples. Fold-change for each receptor upon treatment with beta-estradiol, where the non-treated samples were considered as calibrator. The data were normalized against *GAPDH* (**A**) and *18S rRNA* (**B**) and were plotted as mean ± SD (*N* = 3). Statistical significance is indicated with asterisks (* 0.01 < *p* < 0.05; ** 0.001 < *p* < 0.01).

Previous studies indicated that beta-estradiol was able to regulate the mRNA levels of estrogen receptors in different tissues [55,56,57]. Therefore, we analyzed the effect of a 24 h treatment with beta-estradiol (10,100, 1000 nM) on the mRNA levels for *ESR1* (estrogen receptor 1), *ESR2* (estrogen receptor 2), *GPR30* (G protein-coupled receptor 30), and *NCOA3* (nuclear receptor coactivator 3) in human myometrial TCs from pregnant uterus samples and normalized the data against the housekeeping genes *GAPDH* (Figure 4A) and *18S rRNA* (Figure 4B).

Considering the untreated cells as calibrator and *GAPDH* as the reference gene, beta-estradiol significantly downregulated *ESR1* (One way ANOVA, F = 31, *p* < 0.01), *ESR2* (One way ANOVA, F = 12, *p* < 0.05), *GPR30* (One way ANOVA, F = 49, *p* < 0.01), and *NCOA3* (One way ANOVA, F = 49, *p* < 0.01) (Figure 4A). The Bonferroni post-hoc analysis indicated that: 100 nM beta-estradiol significantly downregulated *ESR1* by 0.55-fold (*p* < 0.05) and *ESR2* by 0.89-fold (*p* < 0.05); 10, 100, and 1000 nM beta-estradiol significantly downregulated *GPR30* by 0.81-fold (*p* < 0.01), 0.97-fold (*p* < 0.01), and 0.72-fold (*p* < 0.01), respectively; 10 and 100 nM beta-estradiol significantly downregulated *NCAO3* by 0.89-fold (*p* < 0.01) and 0.96-fold (*p* < 0.01), respectively, versus the non-treated samples (Figure 4A).

Considering the untreated cells as calibrator and *18S rRNA* as the reference gene, *ESR1* (One way ANOVA, F = 27, *p* < 0.01), *ESR2* (One way ANOVA, F = 14, *p* < 0.05), *GPR30* (One way ANOVA, F = 46, *p* < 0.01), and *NCOA3* (One way ANOVA, F = 92, *p* < 0.001) were downregulated (Figure 4B). The Bonferroni post-hoc analysis indicated that: 100 nM beta-estradiol significantly downregulated *ESR1* by 0.37-fold (*p* < 0.05) and *ESR2* by 0.46-fold (*p* < 0.05); 10, 100, and 1000 nM beta-estradiol significantly downregulated *GPR30* by 0.8-fold (*p* < 0.01), 0.96-fold (*p* < 0.01), and 0.78-fold (*p* < 0.01), respectively; 10 and 100 nM beta-estradiol significantly downregulated *NCAO3* by 0.87-fold (*p* < 0.01) and 0.95-fold (*p* < 0.01), respectively, versus the non-treated samples (Figure 4B).

### 2.5. Beta-Estradiol Partly Blocks Bay K8644-Induced Calcium Transients in Human Myometrial Uterine TCs

**Figure 5:**
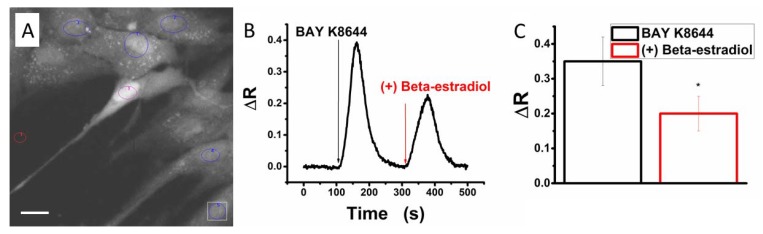
Beta-estradiol effect on voltage-gated calcium channels in TCs from pregnant uterine myometrium by Fura-2-based calcium imaging. (**A**) Cells in primary culture of human uterine myometrium loaded with Fura-2AM. The regions of interest (ROIs) are marked with circles corresponding to: red (reference field without cells), violet (TC), blue (other cells); Scale bar 20 μm (**B**) representative traces of fluorescence ratio (ΔR) changes induced by Bay K8644 (10 μM) in the absence and presence of beta-estradiol (1000 nM) in human myometrial TCs during calcium imaging recordings; (**C**) mean fluorescence ratio (ΔR) ± SD, *N* = 5, in the same conditions as (**B**). The statistical analysis was done by the paired Student’s *t* test, and the level of significance is indicated with asterisks (* *p* < 0.05).

Bay K8644 was demonstrated to specifically activate HVA calcium channels [46]. We applied this agonist in order to test the functional activation of voltage-gated calcium channels in human myometrial TCs from pregnant myometrium by Fura-2AM calcium imaging (see loaded cells in Figure 5A). Consequently, we recorded calcium transients activated by 5 μM Bay K8644 (Figure 5B).

Previous studies indicated that beta-estradiol counteracts the activation exerted by Bay K8644 on cardiac calcium channels [58] and on rat cortical neurons [12,13,59]. In particular, 1000 nM beta-estradiol inhibited the HVA but not the LVA calcium currents in rat sensory neurons via a non-genomic mechanism [12,13,59]. The next step was to test the effect of beta-estradiol (1000 nM) on the calcium transients elicited by Bay K8644 (5 μM) in human myometrial TCs from pregnant uterine samples. Indeed, we evidenced the same partial inhibitory effect of beta-estradiol on calcium influx through voltage-gated calcium channels in TCs (Figure 5B). The mean fluorescence ratio of the calcium signal induced by Bay K8644 was significantly diminished from 0.35 ± 0.07 (in the absence of beta-estradiol) to 0.20 ± 0.05, *N* = 5, *p* < 0.05, paired Student’s *t* test (in the presence of beta-estradiol) Figure 5C.

### 2.6. Beta-Estradiol Partly Inhibits Bay K8644-Induced Calcium Current in Human Myometrial Uterine TCs

**Figure 6:**
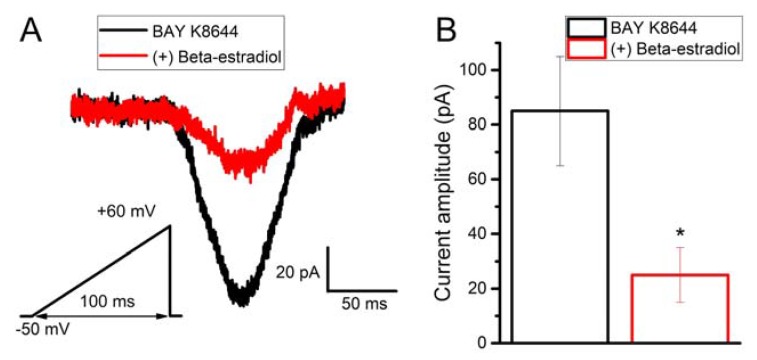
Beta-estradiol partly blocks voltage-gated calcium currents in TCs from pregnant uterine myometrium. (**A**) Representative patch-clamp recording in voltage-clamp mode on a human myometrial TC. Brief depolarizing ramp protocols from −50 to +60 mV with a duration of 100 ms were applied. HVA currents were elicited by perfusion with Bay K8644 (2.5 μM) for 1 min followed by the ramp protocol. To block the calcium current, beta-estradiol (1000 nM) was applied in the presence of Bay K8644; (**B**) amplitude of the voltage-gated calcium currents induced by Bay K8644 in the presence or absence of beta-estradiol. The statistical analysis was done by the paired Student’s *t* test, and the level of significance is indicated with asterisks (* *p* < 0.05).

We have also demonstrated that Bay K8644 was able to activate calcium currents in human myometrial uterine TCs (Figure 6A). The membrane capacitance had a mean of 115 ± 11 pF. Moreover, by adding beta-estradiol (1000 nM) in the presence of Bay K8644, we were able to partly inhibit Bay K8644-induced calcium current (Figure 6A). Beta-estradiol blocked Bay K8644-induced current from 85.12 ± 20.03 to 25.91 ± 10.34 pA, *N* = 6, *p* < 0.05, paired Student’s *t*-test (Figure 6B). This inhibitory effect was present in all human myometrial uterine TCs in which Bay K8644 activated voltage-gated calcium currents.

## 3. Discussion

### 3.1. Beta-Estradiol Regulates Voltage-Gated Calcium Channels in Human Myometrial TCs from Pregnant Uterus

We have previously evidenced HVA calcium currents induced by a brief ramp depolarization protocol in TCs from non-pregnant uterine myometrium [35]. In this study, we obtained Bay K8644-induced currents in TCs from pregnant uterine myometrium with lower amplitude compared to those recorded in TCs from non-pregnant uterine myometrium. This difference might be due to the pregnancy condition and is correlated with the lower expression of *CACNA1C* mRNA (without statistical significance) in pregnant versus non-pregnant uterine samples. While immunofluorescence indicated that the protein expression of the LVA calcium channels (e.g., Cav3.1 and Cav3.2) was upregulated in human myometrial TCs from pregnant versus non-pregnant uterine samples [35], this study showed that gene expression was upregulated for Cav3.1 and downregulated for Cav3.2 and Cav3.3.

Previous reports demonstrated that beta-estradiol regulates the mRNA expression of T-type calcium channel subunits, but the up- or downregulation was dependent on the type of tissue. To date, in the medial preoptic area and the arcuate nucleus, beta-estradiol has been shown to upregulate Cav3.1 and Cav3.2, but not Cav3.3 [60]. Meanwhile, in the pituitary, beta-estradiol has been shown to upregulate Cav3.1 and downregulate Cav3.2 and Cav3.3 [60]. By comparison, our study on human pregnant myometrial TCs indicated that chronic beta-estradiol treatment downregulated Cav3.1, Cav3.2, Cav3.3, and Cav1.2 at higher concentrations. We have also observed that the mRNA encoding Cav3.1 was upregulated in myometrial TCs in the untreated pregnant samples compared to the chronically estrogen-treated pregnant samples. Possible explanations might include the absence of some physiological factors in our preparations, including the bursting release of hormones during pregnancy and labor compared to the continuous chronic exposure (24 h) to estrogen, and the complexity and the interplay of other steroid hormones (e.g., progesterone, oxytocin etc.), prostaglandins, cytokines, nitric oxide, released in parturition [61].

Bay K8644 is a well-known agonist of the L-type calcium channels [38]. Previous studies demonstrated that beta-estradiol (1 μM) inhibited HVA, but not LVA, calcium currents in rat sensory neurons via a non-genomic mechanism [12,13,59]. This inhibition was done in a rapid, reversible and concentration-dependent manner and determined the hyperpolarization shift of the steady-state inactivation curve [12]. Our data are in accordance with these studies and demonstrate the blocking effect of beta-estradiol (1 μM) on high-voltage-activated calcium currents (Bay K8644-activated currents) in TCs from human uterine myometrium. However, 10-fold higher concentrations of beta-estradiol (10 μM) reduced not only the HVA Ba^2+^ current but also the T-type Ca^2+^ current in vascular smooth muscle cells [14]. This effect might be attributed to a non-specific antagonistic effect of beta-estradiol at higher concentrations on both types of voltage-gated calcium channels.

Interestingly, we should mention that beta-estradiol exerted different functional modulatory action on the HVA channels upon acute (1 min) or chronic (24 h) exposure. Thus, acute exposure determined a reduction in the current amplitude by partly antagonizing BAY K8644, while chronic exposure triggered gene expression downregulation.

It is of particular interest to understand if beta-estradiol regulates voltage-gated calcium channels by means of estrogen receptors activation. On this topic, there are controversial reports in the literature regarding the regulation of L-type calcium channels upon beta-estradiol treatment via estrogen receptors. To date, beta-estradiol was demonstrated to inhibit HVA calcium currents in rat cortical neurons without involving the estrogen receptors [12]. In the hypothalamus, beta-estradiol-induced upregulation in Cav3.1 was dependent on ERα, while its effect on Cav3.2 was dependent on both ERα and ERβ [60]. In the pituitary, beta-estradiol-induced effects were only dependent on the expression of ERα [60]. In our study, we did not evaluate if the beta-estradiol effect on voltage-gated calcium channels was mediated by the estrogen receptors. However, corroborating the modulatory effect of beta-estradiol exerted on voltage-gated calcium channels and on estrogen receptors, we might consider that there is an interplay between these key players that involves the activation of calcium signaling pathways. Other hormones (e.g., progesterone, oxytocin) have also been described to block voltage-gated calcium channels [62,63,64], and we might suppose a similar inhibition in TCs human myometrial preparations, but our study was focused only on estrogen effects.

### 3.2. Beta-Estradiol Regulates Estrogen Receptors in Human Myometrial TCs from Pregnant Uterus

Estrogens act specifically on estrogen receptors and mediate distinct roles in pregnancy and labor. Indeed, ERα plays a more prominent role than ERβ in mediating estrogen action in the induction of uterine oxytocin receptors before labor [65]. Alterations of the estrogen–estrogen receptors signaling have been associated with different pathologies of the reproductive system (e.g., preeclampsia, endometrial cancer, etc.). For example, low GPR30 expression levels, increased apoptosis, and reduced proliferation were associated with preeclampsia, and the pharmacological targeting of GPR30 might have clinical relevance [66]. Estrogen-induced PI3K–AKT signaling activated by GPR30 is involved in the regulation of endometrial cancer cell proliferation [67].

Previous studies on human pregnant myometrium samples indicated the expression of ERα and GPR30 mRNAs, while ERβ mRNA was not detectable [53]. By comparison, we retrieved ERα and GPR30 mRNAs expression in TCs from human pregnant myometrium samples, but we also detected low levels of ERβ mRNA. These findings are in accordance with our previous immunocytochemical studies on TCs cultured from human myometrium during and outside pregnancy [28,68]. Beside estrogen receptors, we also demonstrated, in an earlier study, the expression of progesterone receptors in TCs from human myometrial cell cultures [28], but an extensive analysis of progesterone receptors expression in pregnant and nonpregnant conditions was beyond the purpose of the present study.

Multiple studies have demonstrated that beta-estradiol regulates the mRNA levels encoding the estrogen receptors in various tissues and species, including mouse Sertoli cells [41], female rat brain [48], rat uterus [69], etc. In particular, 3 and 6 h after beta-estradiol subcutaneous injection, the downregulation of ERα and ERβ mRNA levels was demonstrated in rat uterus [69]. We showed that beta-estradiol downregulates ESR1, ESR2, GPR30, and NCOA3 in TCs from human pregnant uterine myometrial cultures. Our current data are in agreement with the in vivo downregulation of these receptors in rat uterus.

NCOA3, also known as steroid receptor coactivator-3 (src-3), was described as being associated with uterine endometrial cancer or other cancers, e.g., breast cancer [70,71]. Despite the great interest for this coregulator, little is known about its physiological role in uterine myometrium [71]. Our study documents for the first time NCOA3 expression in uterine TCs and its downregulation in pregnancy and upon beta-estradiol treatment.

Calcium was demonstrated to play an important role in the activation of estrogen receptors in different cell types. Interestingly, stimuli inducing the release of intracellular calcium determine the recruitment of estrogen receptors 1 and stimulate the expression of estrogen-responsive genes [72]. Several studies indicated that beta-estradiol regulates the expression of different receptors associated with calcium signaling mechanisms via estrogen receptors. In mouse N2A and human SK-N-SH neural cells, beta-estradiol upregulated the expression of the α and β subunits of BK channels via estrogen receptor β, in a concentration-dependent manner [10]. Alterations of calcium homeostasis may determine breast cancer progression by affecting various signaling pathways including estrogen receptors [73]. Both intra- and extracellular calcium was shown to influence ERα transcriptional activity in breast cancer cells [74].

In pregnancy, beta-estradiol exerts tissue-dependent regulatory actions. In particular, beta-estradiol and estrogens-specific agonists have been shown to differentially modulate the tone of uterine versus placental arteries, and their contribution to the regulation of human uteroplacental blood flow might be tissue-specific [75]. In rats ovariectomized on day 18 of pregnancy, estrogen treatment caused upregulation of oxytocin receptors [65].

In conclusion, our study brings novel highlights into the modulatory effects of beta-estradiol on the uterine myometrium and shows that voltage-gated calcium channels and estrogen receptors expressed in TCs are essential regulatory targets.

## 4. Materials and Methods

### 4.1. Human Uterine Samples

The myometrial tissue of non-pregnant women was obtained from a total of 10 menstruating, parous, pre-menopausal women during the proliferative phase, which were undergoing hysterectomy for benign gynecological reasons. The median age of the non-pregnant women was 42 years (range 35–49 years). The inclusion criteria used in the selection of the research subjects were indications for surgery for menometrorrhagia/dysmenorrhea as a consequence of localized or diffuse leiomyomas and no other associated medical conditions or treatment history. Hysterectomized uteri were biopsied in the pathology department where they were examined macroscopically, and apparently normal myometrial areas were chosen. The pregnant women included in the study (*n* = 8) were aged between 30 and 35 years, and the gestational age was between 38 and 40 weeks. The patients underwent cesarean surgery in labor because of dystocia and fetal distress. A small strip of myometrium was carefully dissected from the upper margin (in the midline) of the lower segment transverse incision of each patient.

All tissue samples were obtained in accordance with the protocol no. 12290 (18.08.2017) approved by the Ethics Committee of Alessandrescu-Rusescu National Institute of Mother and Child Health. The patients included in the study were enrolled from the Department of Obstetrics and Gynecology, “Polizu” Clinical Hospital, Alessandrescu-Rusescu National Institute of Mother and Child Health, Bucharest, Romania. All patients donating uterine tissue samples that were included in the study signed the informed written consent. The inclusion criteria requested that none of the patients included in the groups received any regular medication for chronic diseases.

### 4.2. Myometrial Cell Cultures

For the qRT-PCR experiments, primary cultures were prepared from human uterine myometrial biopsies as previously described [35]. On the basis of the immunopositivity for CD34 and platelet-derived growth factor receptor-α (PDGFRα) that was already described in human uterine myometrial TCs [35], we employed a double-labeling technique, using goat polyclonal anti-CD34 (#sc-7045, Santa Cruz Biotechnology, Santa Cruz, CA, USA) and rabbit polyclonal anti-PDGFRα (#sc-338, Santa Cruz Biotechnology, USA) antibodies. The primary antibodies were detected by a secondary goat anti-rabbit antibody conjugated to AlexaFluor 488 and a donkey anti-goat antibody conjugated to AlexaFluor 546, from Invitrogen Molecular Probes, Eugene, OR, USA. An enriched TC culture (CD34^+^/PDGFRα^+^ cells) was obtained by sorting the double-labeled cells using a BD FACSCanto II - Becton Dickinson flow cytometer (BD Biosciences, Waltham, MA, USA), as previously described for cardiac TCs [69]. For the qRT-PCR experiments, the cells were chronically exposed for 24 h to beta-estradiol (#E2758, Sigma-Aldrich, St. Louis, MO, USA) at concentrations of 10, 100, and 1000 nM in serum-free medium, in order to avoid the non-specific binding of beta-estradiol to the proteins in the serum.

For the patch-clamp and calcium imaging experiments, primary cultures were prepared from human uterine myometrial biopsies as previously described [35]. The cells were used without further sorting in order to maintain the physiological environment and to prevent any alteration in the calcium signaling due to the absence of human myometrial muscle cells in the cell culture. The human myometrial cells (between first and fourth passages) were plated at a density of 5 × 10^4^ cells/cm^2^ on 24 mm Petri dishes for the patch-clamp experiments or on 24 mm glass coverslips for the calcium imaging experiments. On the basis of their morphological features (e.g., long and moniliform telopodes), only TCs were selected under the microscope for patch-clamp or calcium imaging recordings. TCs were acutely exposed for 1 min to beta-estradiol in the patch-clamp and calcium-imaging experiments, in the presence or absence of BAY K8644.

### 4.3. qRT-PCR

To quantify the mRNA expression levels encoding for different genes (Table 1) in primary cell cultures from human uterine myometrium, total RNA was extracted using the GenElute Mammalian Total RNA MiniPrep Kit (RTN70, Sigma), according to the manufacturer’s instructions. RNA concentrations were determined by spectrophotometric measurements of absorption at 260 and 280 nm (Beckman Coulter DU 730, Carlsbad, CA, USA), and DNase I treatment was applied in order to remove contaminating genomic DNA. In agreement with the manufacturer’s guidelines (Sigma-Aldrich, USA), in our experiments, the A260:A280 ratio was 2.04 ± 0.05. Reverse transcription was done using the High-Capacity cDNA Archive Kit (Applied Biosystems, Waltham, MA, USA). The human primers and TaqMan probes (Life Technologies, Carlsbad, CA, USA) used in our experiments are listed in Table 1 and were used in accordance with the manufacturer’s guidelines. *GAPDH* and *18S rRNA* were used as reference genes. The relative abundance of gene transcripts was assessed via qRT-PCR, using the TaqMan methodology and the ABI Prism 7300 Sequence Detection System (Applied Biosystems, USA). The reactions were carried out in triplicate for 50 cycles.

### 4.4. Intracellular Calcium Imaging

Human myometrial TCs plated on 24 mm coverglasses were incubated for 45 min at room temperature in a dark chamber with 4 μM Fura-2 acetoxymethyl ester (Fura-2 AM; #F1221, Thermo Fisher Scientific, Waltham, MA, USA) and 0.25% pluronic Pluronic™ F-127 (#P3000MP, Thermo Fisher Scientific, USA) in Dulbecco’s Modified Eagle’s Medium (DMEM; Thermo Fisher Scientific, USA). The cells were rinsed three times with Ringer solution (in mM: NaCl 126, HEPES 5, CaCl_2_ 2, MgCl_2_ 2, glucose 10, pH 7.4 (with Tris base), let recover for 15 min, and then imaged with an IX-71 Olympus microscope. The excitation was performed by a Xe lamp with monochromator Polychrome V (Till Photonics GmbH, Gräfelfing, Germany) at 340 ± 5 and 380 ± 5 nm, while the emission was collected by a filter at 510 ± 20 nm. The images were acquired by a cooled CCD camera iXON+ EM DU 897 (Andor, Belfast, Northern Ireland) controlled by iQ 1.8 software package, with a frequency of one pair of images per 2 s. Ca^2+^ transients were triggered by 1 min application of 5 μM (±)-Bay K8644 (#B112, Sigma-Aldrich, St. Louis, MO, USA), an L-type calcium channel agonist [46]. Beta-estradiol was applied for 1 min at 1000 nM in the presence of Bay K8644, and Ca^2+^ transients were recorded. The perfusion was performed with an MPS-2 system (World Precision Instruments, Sarasota, FL, USA). Each coverglass bearing human myometrial TCs was used for a single experimental variant.

### 4.5. Patch-Clamp Recordings on TCs

TCs from pregnant uterus were recorded in whole-cell configuration under the voltage-clamp mode, using an AxoPatch 200B amplifier (Molecular Devices, San Jose, CA, USA). The electrodes were pulled from borosilicate glass capillaries (GC150F; Harvard Apparatus, Edenbridge, Kent, UK) and heat polished. The final resistance of the pipette, when filled with internal solution, was 3–4 MΩ. The perfusion was performed with an MPS-2 (World Precision Instruments, Sarasota, FL, USA) system, with the tip placed at approximately 100 μm from the cell. Membrane currents were low-pass filtered at 3 kHz (−3 dB, three pole Bessel) and sampled with an Axon Digidata 1440 data acquisition system (Molecular Devices, USA), using pClamp 10 software in gap-free mode. All electrophysiological experiments were performed at room temperature (25 °C). The bath and pipette solutions were used as previously described [35,76]. We adapted the previously described protocol [35] and we applied brief depolarizing ramp protocols from −50 to +60 mV with a duration of 100 ms in order to elicit HVA currents. Uterine TCs were perfused with (±)-Bay K8644 (#B112, Sigma-Aldrich, USA) for 1 min at 2.5 µM concentration. Beta-estradiol was applied for 1 min at 1000 nM in the presence of Bay K8644.

### 4.6. Data Analysis

Quantitative RT-PCR data were obtained by normalizing the mRNA levels encoding for different genes to those of *GAPDH* mRNA level using the 2(-Delta C(T)) method, as previously described [77]. Statistical analysis comparing the mRNA expression for each subtype of voltage-gated calcium channel relative to the reference gene (*GAPDH* or *18S rRNA*) in pregnant and in non-pregnant myometrial TCs, was carried out by one-way ANOVA analysis followed by post-hoc Bonferroni test. The effect of pregnancy on the mRNA levels of the voltage-gated calcium channels or the estrogen receptors was analyzed by considering the unpregnant samples as calibrator and performing the one-way ANOVA analysis followed by the post-hoc Bonferroni test. The beta-estradiol effect on the mRNA levels of the voltage-gated calcium channels or the estrogen receptors was analyzed by considering the non-treated samples as calibrator and performing the one-way ANOVA analysis followed by the post-hoc Bonferroni test.

Quantitative calcium imaging results were expressed by means of emission ratio R = I_340_/I_380_, I being the emission intensity for excitation at 340 and 380 nm, which is proportional to the free cytosolic Ca^2+^ concentration ([Ca^2+^]_i_). The mean fluorescence ratios (ΔR) of the calcium transients induced by BAY K8644 in the absence or presence of beta-estradiol were compared by unpaired Student’s *t* test.

The amplitudes of the HVA calcium currents recorded by patch-clamp in the absence or presence of beta-estradiol were compared by unpaired Student’s *t* test.

All data analysis and data plotting were performed using OriginPro 8 (OriginLab Corporation, Northampton, MA, USA).

## Figures and Tables

**Table 1:** Panel of primers (Life Technologies, Carlsbad, CA, USA).

Primer	Gene	Protein Encoded
Hs00167681_m1	*CACNA1C*	Cav1.2, L-type calcium channel
Hs00367969_m1	*CACNA1G*	Cav3.1, T-type calcium channel
Hs00234934_m1	*CACNA1H*	Cav3.2, T-type calcium channel
Hs00184168_m1	*CACNA1I*	Cav3.3, T-type calcium channel
Hs01922715_s1	*GPR30, GPER1*	G protein-coupled estrogen receptor 1
Hs01105253_m1	*NCOA3*	Nuclear receptor coactivator 3
Hs01100353_m1	*ESR2*	Estrogen receptor 2
Hs00174860_m1	*ESR1*	Estrogen receptor 1
Hs99999905_m1	*GAPDH*	Glyceraldehyde 3-phosphate dehydrogenase
Hs99999901_s1	*18S rRNA*	18S ribosomal RNA

## References

[1] Wray S., Jones K., Kupittayanant S., Li Y., Matthew A., Monir-Bishty E., Noble K., Pierce S.J., Quenby S., Shmygol A.V. (2003). Calcium signaling and uterine contractility. J. Soc. Gynecol. Investig..

[2] Herington J.L., Swale D.R., Brown N., Shelton E.L., Choi H., Williams C.H., Hong C.C., Paria B.C., Denton J.S., Reese J. (2015). High-Throughput Screening of Myometrial Calcium-Mobilization to Identify Modulators of Uterine Contractility. PLoS ONE.

[3] Bernstein K., Vink J.Y., Fu X.W., Wakita H., Danielsson J., Wapner R., Gallos G. (2014). Calcium-activated chloride channels anoctamin 1 and 2 promote murine uterine smooth muscle contractility. Am. J. Obstet. Gynecol..

[4] Pistilli M.J., Petrik J.J., Holloway A.C., Crankshaw D.J. (2012). Immunohistochemical and functional studies on calcium-sensing receptors in rat uterine smooth muscle. Clin. Exp. Pharmacol. Physiol..

[5] Ying L., Becard M., Lyell D., Han X., Shortliffe L., Husted C.I., Alvira C.M., Cornfield D.N. (2015). The transient receptor potential vanilloid 4 channel modulates uterine tone during pregnancy. Sci. Transl. Med..

[6] Wakle-Prabagaran M., Lorca R.A., Ma X., Stamnes S.J., Amazu C., Hsiao J.J., Karch C.M., Hyrc K.L., Wright M.E., England S.K. (2016). BKCa channel regulates calcium oscillations induced by alpha-2-macroglobulin in human myometrial smooth muscle cells. Proc. Natl. Acad. Sci. USA.

[7] Wray S. (2015). Insights from physiology into myometrial function and dysfunction. Exp. Physiol..

[8] Wray S., Burdyga T., Noble D., Noble K., Borysova L., Arrowsmith S. (2015). Progress in understanding electro-mechanical signalling in the myometrium. Acta Physiol..

[9] Lee S.E., Ahn D.S., Lee Y.H. (2009). Role of T-type Ca Channels in the Spontaneous Phasic Contraction of Pregnant Rat Uterine Smooth Muscle. Korean J. Physiol. Pharmacol..

[10] Li X.T., Qiu X.Y. (2015). 17β-Estradiol Upregulated Expression of α and β Subunits of Larger-Conductance Calcium-Activated K(+) Channels (BK) via Estrogen Receptor β. J. Mol. Neurosci..

[11] Rahbek M., Nazemi S., Odum L., Gupta S., Poulsen S.S., Hay-Schmidt A., Klaerke D.A. (2014). Expression of the small conductance Ca^2+^-activated potassium channel subtype 3 (SK3) in rat uterus after stimulation with 17β-estradiol. PLoS ONE.

[12] Wang Q., Ye Q., Lu R., Cao J., Wang J., Ding H., Gao R., Xiao H. (2014). Effects of estradiol on high-voltage-activated Ca(2+) channels in cultured rat cortical neurons. Endocr. Res..

[13] Sánchez J.C., López-Zapata D.F., Pinzón O.A. (2014). Effects of 17beta-estradiol and IGF-1 on L-type voltage-activated and stretch-activated calcium currents in cultured rat cortical neurons. Neuroendocrinol. Lett..

[14] Zhang F., Ram J.L., Standley P.R., Sowers J.R. (1994). 17 beta-Estradiol attenuates voltage-dependent Ca^2+^ currents in A7r5 vascular smooth muscle cell line. Am. J. Physiol..

[15] Sánchez J.C., López-Zapata D.F., Francis L., De Los Reyes L. (2011). Effects of estradiol and IGF-1 on the sodium calcium exchanger in rat cultured cortical neurons. Cell. Mol. Neurobiol..

[16] Choi Y., Seo H., Kim M., Ka H. (2009). Dynamic expression of calcium-regulatory molecules, TRPV6 and S100G, in the uterine endometrium during pregnancy in pigs. Biol. Reprod..

[17] Pohóczky K., Kun J., Szalontai B., Szőke É., Sághy É., Payrits M., Kajtár B., Kovács K., Környei J.L., Garai J. (2016). Estrogen-dependent up-regulation of TRPA1 and TRPV1 receptor proteins in the rat endometrium. J. Mol. Endocrinol..

[18] Tulchinsky D., Korenman S.G. (1971). The plasma estradiol as an index of fetoplacental function. J. Clin. Investig..

[19] Hatthachote P., Gillespie J.I. (1999). Complex interactions between sex steroids and cytokines in the human pregnant myometrium: Evidence for an autocrine signaling system at term. Endocrinology.

[20] Wu J.J., Geimonen E., Andersen J. (2000). Increased expression of estrogen receptor beta in human uterine smooth muscle at term. Eur. J. Endocrinol..

[21] Cretoiu S.M., Cretoiu D., Popescu L.M. (2012). Human myometrium—The ultrastructural 3D network of telocytes. J. Cell. Mol. Med..

[22] Cretoiu S.M., Popescu L.M. (2014). Telocytes revisited. Biomol. Concepts.

[23] Song D., Cretoiu D., Cretoiu S.M., Wang X. (2016). Telocytes and lung disease. Histol. Histopathol..

[24] Cretoiu S.M., Cretoiu D., Marin A., Radu B.M., Popescu L.M. (2013). Telocytes: Ultrastructural, immunohistochemical and electrophysiological characteristics in human myometrium. Reproduction.

[25] Song D., Cretoiu D., Zheng M., Qian M., Zhang M., Cretoiu S.M., Chen L., Fang H., Popescu L.M., Wang X. (2016). Comparison of Chromosome 4 gene expression profile between lung telocytes and other local cell types. J. Cell. Mol. Med..

[26] Albulescu R., Tanase C., Codrici E., Popescu D.I., Cretoiu S.M., Popescu L.M. (2015). The secretome of myocardial telocytes modulates the activity of cardiac stem cells. J. Cell. Mol. Med..

[27] Zheng Y., Cretoiu D., Yan G., Cretoiu S.M., Popescu L.M., Fang H., Wang X. (2014). Protein profiling of human lung telocytes and microvascular endothelial cells using iTRAQ quantitative proteomics. J. Cell. Mol. Med..

[28] Cretoiu S.M., Cretoiu D., Simionescu A., Popescu L.M., Kahn S. (2012). Telocytes in human fallopian tube and uterus express estrogen and progesterone receptors. Sex Steroids.

[29] Roatesi I., Radu B.M., Cretoiu D., Cretoiu S.M. (2015). Uterine Telocytes: A Review of Current Knowledge. Biol. Reprod..

[30] Cretoiu D., Xu J., Xiao J., Cretoiu S.M. (2016). Telocytes and Their Extracellular Vesicles-Evidence and Hypotheses. Int. J. Mol. Sci..

[31] Cretoiu D., Cretoiu S.M. (2016). Telocytes in the reproductive organs: Current understanding and future challenges. Semin. Cell Dev. Biol..

[32] Cretoiu D., Radu B.M., Banciu A., Banciu D.D., Cretoiu S.M. (2017). Telocytes heterogeneity: From cellular morphology to functional evidence. Semin. Cell Dev. Biol..

[33] Rusu M.C., Cretoiu D., Vrapciu A.D., Hostiuc S., Dermengiu D., Manoiu V.S., Cretoiu S.M., Mirancea N. (2016). Telocytes of the human adult trigeminal ganglion. Cell Biol. Toxicol..

[34] Radu B.M., Banciu A., Banciu D.D., Radu M., Cretoiu D., Cretoiu S.M. (2017). Calcium Signaling in Interstitial Cells: Focus on Telocytes. Int. J. Mol. Sci..

[35] Cretoiu S.M., Radu B.M., Banciu A., Banciu D.D., Cretoiu D., Ceafalan L.C., Popescu L.M. (2015). Isolated human uterine telocytes: Immunocytochemistry and electrophysiology of T-type calcium channels. Histochem. Cell Biol..

[36] Campeanu R.A., Radu B.M., Cretoiu S.M., Banciu D.D., Banciu A., Cretoiu D., Popescu L.M. (2014). Near-infrared low-level laser stimulation of telocytes from human myometrium. Lasers Med. Sci..

[37] Othman E.R., Elgamal D.A., Refaiy A.M., Abdelaal I.I., Abdel-Mola A.F., Al-Hendy A. (2016). Identification and potential role of telocytes in human uterine leiomyoma. Contracept. Reprod. Med..

[38] Varga I., Klein M., Urban L., Danihel L., Polak S., Danihel L. (2018). Recently discovered interstitial cells “telocytes” as players in the pathogenesis of uterine leiomyomas. Med. Hypotheses.

[39] Cretoiu S.M. (2016). Immunohistochemistry of Telocytes in the Uterus and Fallopian Tubes. Adv. Exp. Med. Biol..

[40] Vannucchi M.G., Faussone-Pellegrini M.S. (2016). The Telocyte Subtypes. Adv. Exp. Med. Biol..

[41] Yang J., Li Y., Xue F., Liu W., Zhang S. (2017). Exosomes derived from cardiac telocytes exert positive effects on endothelial cells. Am. J. Transl. Res..

[42] Rusu M.C., Hostiuc S., Vrapciu A.D., Mogoantă L., Mănoiu V.S., Grigoriu F. (2017). Subsets of telocytes: Myocardial telocytes. Ann. Anat..

[43] Uluer E.T., Inan S., Ozbilgin K., Karaca F., Dicle N., Sancı M. (2015). The role of hypoxia related angiogenesis in uterine smooth muscle tumors. Biotech. Histochem..

[44] Sajewicz M., Konarska M., Wrona A.N., Aleksandrovych V., Bereza T., Komnata K., Solewski B., Maleszka A., Depukat P., Warchoł Ł. (2016). Vascular density, angiogenesis and pro-angiogenic factors in uterine fibroids. Folia Med. Cracov..

[45] Salama N. (2013). Immunohistochemical characterization of telocytes in rat uterus in different reproductive states. Egypt J. Histol..

[46] Kim Y.H., Chung S., Lee Y.H., Kim E.C., Ahn D.S. (2012). Increase of L-type Ca^2+^ current by protease-activated receptor 2 activation contributes to augmentation of spontaneous uterine contractility in pregnant rats. Biochem. Biophys. Res. Commun..

[47] Seda M., Pinto F.M., Wray S., Cintado C.G., Noheda P., Buschmann H., Candenas L. (2007). Functional and molecular characterization of voltage-gated sodium channels in uteri from nonpregnant rats. Biol. Reprod..

[48] Tica A.A., Dun E.C., Tica O.S., Gao X., Arterburn J.B., Brailoiu G.C., Oprea T.I., Brailoiu E. (2011). G protein-coupled estrogen receptor 1-mediated effects in the rat myometrium. Am. J. Physiol. Cell Physiol..

[49] Silva E.S., Scoggin K.E., Canisso I.F., Troedsson M.H., Squires E.L., Ball B.A. (2014). Expression of receptors for ovarian steroids and prostaglandin E2 in the endometrium and myometrium of mares during estrus, diestrus and early pregnancy. Anim. Reprod. Sci..

[50] Kautz E., Gram A., Aslan S., Ay S.S., Selçuk M., Kanca H., Koldaş E., Akal E., Karakaş K., Findik M. (2014). Expression of genes involved in the embryo-maternal interaction in the early-pregnant canine uterus. Reproduction.

[51] Ilicic M., Butler T., Zakar T., Paul J.W. (2017). The expression of genes involved in myometrial contractility changes during ex situ culture of pregnant human uterine smooth muscle tissue. J. Smooth Muscle Res..

[52] Vodstrcil L.A., Shynlova O., Westcott K., Laker R., Simpson E., Wlodek M.E., Parry L.J. (2010). Progesterone withdrawal, and not increased circulating relaxin, mediates the decrease in myometrial relaxin receptor (RXFP1) expression in late gestation in rats. Biol. Reprod..

[53] Welsh T., Johnson M., Yi L., Tan H., Rahman R., Merlino A., Zakar T., Mesiano S. (2012). Estrogen receptor (ER) expression and function in the pregnant human myometrium: Estradiol via ERα activates ERK1/2 signaling in term myometrium. J. Endocrinol..

[54] Chandran S., Cairns M.T., O’Brien M., Smith T.J. (2014). Transcriptomic effects of estradiol treatment on cultured human uterine smooth muscle cells. Mol. Cell. Endocrinol..

[55] Lin J., Zhu J., Li X., Li S., Lan Z., Ko J., Lei Z. (2014). Expression of genomic functional estrogen receptor 1 in mouse sertoli cells. Reprod. Sci..

[56] Yamaguchi N., Yuri K. (2014). Estrogen-dependent changes in estrogen receptor-β mRNA expression in middle-aged female rat brain. Brain Res..

[57] Murata T., Narita K., Ichimaru T. (2014). Rat uterine oxytocin receptor and estrogen receptor α and β mRNA levels are regulated by estrogen through multiple estrogen receptors. J. Reprod..

[58] Bechem M., Hoffmann H. (1993). The molecular mode of action of the Ca agonist (-) BAY K 8644 on the cardiac Ca channel. Pflugers Arch..

[59] Lee D.Y., Chai Y.G., Lee E.B., Kim K.W., Nah S.Y., Oh T.H., Rhim H. (2002). 17Beta-estradiol inhibits high-voltage-activated calcium channel currents in rat sensory neurons via a non-genomic mechanism. Life Sci..

[60] Bosch M.A., Hou J., Fang Y., Kelly M.J., Rønnekleiv O.K. (2009). 17Beta-estradiol regulation of the mRNA expression of T-type calcium channel subunits: Role of estrogen receptor alpha and estrogen receptor beta. J. Comp. Neurol..

[61] Ravanos K., Dagklis T., Petousis S., Margioula-Siarkou C., Prapas Y., Prapas N. (2015). Factors implicated in the initiation of human parturition in term and preterm labor: A review. Gynecol. Endocrinol..

[62] Luoma J.I., Kelley B.G., Mermelstein P.G. (2011). Progesterone inhibition of voltage-gated calcium channels is a potential neuroprotective mechanism against excitotoxicity. Steroids.

[63] Sun J., Moenter S.M. (2010). Progesterone treatment inhibits and dihydrotestosterone (DHT) treatment potentiates voltage-gated calcium currents in gonadotropin-releasing hormone (GnRH) neurons. Endocrinology.

[64] Liu B., Hill S.J., Khan R.N. (2005). Oxytocin inhibits T-type calcium current of human decidual stromal cells. J. Clin. Endocrinol. Metab..

[65] Murata T., Narita K., Honda K., Matsukawa S., Higuchi T. (2003). Differential regulation of estrogen receptor alpha and beta mRNAs in the rat uterus during pregnancy and labor: Possible involvement of estrogen receptors in oxytocin receptor regulation. Endocr. J..

[66] Li J., Chen Z., Zhou X., Shi S., Qi H., Baker P.N., Zhang H. (2016). Imbalance between proliferation and apoptosis-related impaired GPR30 expression is involved in preeclampsia. Cell Tissue Res..

[67] Wei Y., Zhang Z., Liao H., Wu L., Wu X., Zhou D., Xi X., Zhu Y., Feng Y. (2012). Nuclear estrogen receptor-mediated Notch signaling and GPR30-mediated PI3K/AKT signaling in the regulation of endometrial cancer cell proliferation. Oncol. Rep..

[68] Cretoiu D., Ciontea S.M., Popescu L.M., Ceafalan L., Ardeleanu C. (2006). Interstitial Cajal-like cells (ICLC) as steroid hormone sensors in human myometrium: Immunocytochemical approach. J. Cell. Mol. Med..

[69] Li Y.Y., Zhang S., Li Y.G., Wang Y. (2016). Isolation, culture, purification and ultrastructural investigation of cardiac telocytes. Mol. Med. Rep..

[70] Sakaguchi H., Fujimoto J., Sun W.S., Tamaya T. (2007). Clinical implications of steroid receptor coactivator (SRC)-3 in uterine endometrial cancers. J. Steroid Biochem. Mol. Biol..

[71] Szwarc M.M., Kommagani R., Lessey B.A., Lydon J.P. (2014). The p160/steroid receptor coactivator family: Potent arbiters of uterine physiology and dysfunction. Biol. Reprod..

[72] Divekar S.D., Storchan G.B., Sperle K., Veselik D.J., Johnson E., Dakshanamurthy S., Lajiminmuhip Y.N., Nakles R.E., Huang L., Martin M.B. (2011). The role of calcium in the activation of estrogen receptor-alpha. Cancer Res..

[73] Tajbakhsh A., Pasdar A., Rezaee M., Fazeli M., Soleimanpour S., Hassanian S.M., FarshchiyanYazdi Z., Younesi Rad T., Ferns G.A., Avan A. (2018). The current status and perspectives regarding the clinical implication of intracellular calcium in breast cancer. J. Cell. Physiol..

[74] Leclercq G. (2012). Calcium-induced activation of estrogen receptor alpha—New insight. Steroids.

[75] Corcoran J.J., Nicholson C., Sweeney M., Charnock J.C., Robson S.C., Westwood M., Taggart M.J. (2014). Human uterine and placental arteries exhibit tissue-specific acute responses to 17β-estradiol and estrogen-receptor-specific agonists. Mol. Hum. Reprod..

[76] Comunanza V., Carbone E., Marcantoni A., Sher E., Ursu D. (2011). Calcium-dependent inhibition of T-type calcium channels by TRPV1 activation in rat sensory neurons. Pflügers Arch. Eur. J. Physiol..

[77] Livak K.J., Schmittgen T.D. (2001). Analysis of relative gene expression data using real-time quantitative PCR and the 2^−∆∆*C*t^. Methods.

